# The photoactivated antifungal activity and possible mode of action of sodium pheophorbide a on *Diaporthe mahothocarpus* causing leaf spot blight in *Camellia oleifera*

**DOI:** 10.3389/fmicb.2024.1403478

**Published:** 2024-06-13

**Authors:** Xu-Long Shi, Jing Yang, Yu Zhang, Piao Qin, He-Ying Zhou, Yun-Ze Chen

**Affiliations:** ^1^College of Forestry, Guizhou University, Guiyang, China; ^2^School of Biological Sciences, Guizhou Education University, Guiyang, China

**Keywords:** sodium pheophorbide a, antifungal activity, *Diaporthe mahothocarpus*, RNA-seq, oxidative stress

## Abstract

**Introduction:**

Sodium pheophorbide a (SPA) is a natural plant-derived photosensitizer, with high photoactivated antifungal activity against some phytopathogenic fungi. However, its fungicidal effect on *Diaporthe mahothocarpus*, a novel pathogen that causes *Camellia oleifera* leaf spot blight, is unclear.

**Methods:**

In the present study, we explored its inhibitory effects on spore germination and mycelial growth of *D. mahothocarpus*. Then we determined its effects on the cell membrane, mycelial morphology, redox homeostasis, and cell death through bioassay. Finally, RNA-seq was used further to elucidate its mode of action at the transcriptional level.

**Results:**

We found that SPA effectively inhibited the growth of *D. mahothocarpus*, with half-maximal effective concentrations to inhibit mycelial growth and spore germination of 1.059 and 2.287 mg/mL, respectively. After 1.0 mg/mL SPA treatment, the conductivity and malondialdehyde content of *D. mahothocarpus* were significantly increased. Scanning electron microscopy and transmission electron microscopy indicated that SPA significantly affected the morphology and ultrastructure of *D. mahothocarpus* hyphae, revealing that SPA can destroy the mycelial morphology and cell structure, especially the cell membrane of *D. mahothocarpus*. Furthermore, transcriptome analysis revealed that SPA significantly suppressed the expression of genes involved in morphology, cell membrane permeability, and oxidative stress. Then, we also found that SPA significantly promoted the accumulation of reactive oxygen species (ROS) in of *D. mahothocarpus*, while it decreased the content of reduced glutathione, inhibited the enzyme activities of superoxide dismutase and catalase, and exacerbated DNA damage. Annexin V-FITC/PI staining also confirmed that 1.0 mg/mL SPA could significantly induce apoptosis and necrosis.

**Discussion:**

Generally, SPA can induce ROS-mediated oxidative stress and cell death, thus destroying the cell membrane and hyphal morphology, and ultimately inhibiting mycelial growth, which indicates that SPA has multiple modes of action, providing a scientific basis for the use of SPA as an alternative plant-derived photoactivated fungicide against *C. oleifera* leaf spot blight.

## 1 Introduction

*Camellia oleifera*, commonly known as tea oil camellia, is a unique woody edible oil tree species widely distributed in southern China (Wu et al., 2022). In recent years, *C. oleifera* production has been vigorously developed in Guizhou Province, reaching a planting area of over 133.6 hm^2^. However, *C. oleifera* production is heavily threatened by diseases, particularly anthracnose caused by *Colletotrichum* species (Li and Li, 2022). During a recent investigation conducted from June to August 2022, a novel new leaf spot blight characterized by irregular brownish-gray lesions was observed on *C. oleifera* in Longli County, Guizhou Province, which caused defoliation and hindered *C. oleifera* growth in severe cases. Based on its morphological and molecular characteristics, we previously identified the pathogen as *Diaporthe mahothocarpus*, belonging to the *D. eres* species complex (Shi et al., 2023).

*Diaporthe*, a complex genus including more than 200 species, is an important plant pathogen genus that can cause diseases in a wide range of host plants, including crops, trees, and ornamental plants (Zhou and Hou, 2019). The diseased plants mainly exhibit symptoms such as ulceration, decay, necrosis, leaf spots, wilting, and withering (Sebastianes et al., 2012). Yang et al. (2021) proposed and described *D. camelliae-oleiferae* and *D. hunanensis* as newly discovered pathogens causing tea oil camellia leaf spots in Hunan Province, China. Diseases caused by *Diaporthe* species are emerging as problematic *Camellia* leaf diseases in southern China, so disease control is a key focus. At present, the prevention and control of diseases occurring in *C. oleifera* is still mainly based on chemical agents. However, prolonged use of conventional chemical fungicides may have significant drawbacks, such as the emergence of infections resistant to fungicides, higher expenses, and possible hazards to the environment and public health (Cools and Hammond-Kosack, 2013; Duraisamy et al., 2022; McLaughlin et al., 2023). Therefore, it is imperative to investigate an effective and environmentally friendly fungicide with low toxicity to responsibly reduce the incidence and spread of leaf spot blight of *C. oleifera* and avoid economic losses in *C. oleifera* production in Guizhou Province.

The emergence of photoactivated pesticides has addressed the shortcomings of chemical pesticides and traditional biopesticides, offering enormous development potential (Braga et al., 2022). Photoactivated pesticides are photosensitizers that can utilize natural factors, such as sunlight and oxygen, in the plant growth environment as excitation and auxiliary conditions that enhance their biological activity (Islam et al., 2022). Under aerobic conditions, the reactive oxygen species (ROS) generated after photosensitizer activation may induce apoptosis or necrosis to trigger cell death of pathogens (Yan et al., 2024). For example, a novel photosensitive iridium complex can induce synergistic iron-induced death and apoptosis to inhibit cancer cells (Yuan et al., 2021). Pheophorbide a (PA), which is created by the breakdown of photosynthetic pigment chlorophyll a, is a natural photosensitizer with multiple action sites and significant photoactivated anti-tumor (Park et al., 2016), antibacterial (Bayarmaa et al., 2009), antiviral (Bouslama et al., 2011), and insecticidal (Singh et al., 2017) activities. Hajri et al. (1999) found that PA inhibits the growth of cancer cells by inducing apoptosis in human pancreatic cancer cells and animal models (nude mice carrying xenotransplanted tumors). Sodium pheophorbide a (SPA) is a water-soluble sodium salt of PA, and it is absorbed by cells faster than PA (Nagai et al., 2014). In our previous research, SPA was demonstrated to inhibit the mycelial growth of some plant pathogenic fungi, including *Fusarium oxysporum*, *Pestalotiopsis neglecta* (Yang et al., 2019), and *Botrytis cinerea* (Ji et al., 2020), so it may have the potential to be developed as a novel plant-based photoactivated fungicide for the prevention and control of *Diaporthe* leaf spot blight on *C. oleifera*. However, the antifungal activity of SPA on *D. mahothocarpus* is not yet clear.

Thus, as a continuation of the described exploratory investigation of the photoactivation mode of SPA, the present study evaluated the photoactivated antifungal activity and mode of action of SPA against *D. mahothocarpus* based on transcriptome sequencing, aiming to provide an empirical basis for the green control of *Diaporthe* leaf spot blight on *C. oleifera*.

## 2 Materials and methods

### 2.1 Plant materials, pathogens, and spore suspensions

Healthy 1-year-old *C. oleifera* seedlings were provided by the Qianyu Oil Tea Seedling Base in Yuping County, Guizhou Province, China. *Diaporthe mahothocarpus* was isolated from leaf spot blight-diseased leaves of *C. oleifera*, cultured on potato dextrose agar (PDA) medium at 28°C, and preserved at 4°C in the Forest Pathology Laboratory of the College of Forestry, Guizhou University, Guiyang, China. Spores were collected by filling a plate of a 7-day-old culture with sterile water and vortexing it for 30 s. The spore suspensions were filtered through sterilized cotton and diluted to 1 × 10^6^ spores/ml.

### 2.2 Reagents

Sodium pheophorbide a (98%) was purchased from Haining Chlorophyll Co., Ltd. (Haining, China). Malondialdehyde (MDA) content, total glutathione (T-GSH)/oxidized glutathione (GSSG) content, and superoxide dismutase (SOD) and catalase (CAT) activity assay kits were purchased from Suzhou Grace Biotechnology Co., Ltd. (Suzhou, China). Soluble protein content, ROS content, Annexin V-FITC/PI double staining apoptosis detection, and DNA damage assay kits were obtained from Nanjing Jiancheng Bioengineering Institute Co., Ltd. (Nanjing, China). Analytical grade reagents and solvents were sourced from Sinopharm Chemical Reagent Co., Ltd. (Shanghai, China).

### 2.3 Photoactivated antifungal activity of SPA against *Diaporthe mahothocarpus*

A previous method was slightly modified to determine the effect of SPA on the mycelial growth of *D. mahothocarpus* (Yang et al., 2019). Mycelial plugs (5.0 mm in diameter) from 7-day-old fungal cultures were placed at the center of 60-mm Petri dishes containing 5 mL of PDA with different concentrations of SPA solution (0.625, 1.25, 2.5, 5.0, 10.0, and 20.0 mg/mL), which were then incubated at 28°C either under light or in the dark. The SPA-containing dishes with fungi plugs were placed in an incubator with built-in light bulbs (4,000 lx). Dishes wrapped with foil were incubated in the dark. After 5 days, mycelial radial growth diameters were measured to calculate the inhibition rates (Jia et al., 2016).

The effect of SPA on the spore germination of *D. mahothocarpus* was assessed according to the method described by Qiu et al. (2014), with a concentration gradient of 0.625, 1.25, 2.5, 5.0, 10.0, and 20.0 mg/mL. The obtained slides were placed in a 28°C incubator with a light intensity of 4,000 lx. The dark control groups were wrapped with foil. After 24 h, the number of germinated spores was recorded to calculate the inhibition rates.

Furthermore, the *in vivo* antifungal effect was investigated in the laboratory. To assess the protective effect of SPA, a volume of 5 mL of different concentrations of SPA (10 and 20 mg/mL) was first sprayed on all the leaves of *C. oleifera* seedlings. After 24 h, each plant was evenly sprayed with 10 mL of spore suspension. To assess the fungal control effect, 10 mL of spore suspension was first sprayed, and 5 mL of SPA was sprayed after 24 h. The control groups in both experiments were sprayed with an equal volume of sterile water. For both experiments, the incidence of leaf spot blight disease was observed and calculated after 15 days.

### 2.4 Effect of SPA on cell membrane permeability and lipid peroxidation

The spore suspensions of *D. mahothocarpus* were inoculated into potato dextrose broth (PDB) medium containing 1.0 mg/mL SPA (the half-maximal effective concentration, EC_50_) and were cultured for 3 days at 28°C in a shaking incubator at 160 rpm under light (4,000 lx). An equal volume of distilled water was added as a control. After incubation, the hyphae and supernatant were separately collected by filtration and then centrifuged at 8,000 rpm for 10 min.

Cell membrane permeability was assessed by extracellular conductivity according to the methods described in a previous study (Yang et al., 2020). A DDS-11A digital conductivity meter (Shanghai INESA Scientific Instrument Co., Ltd., Shanghai, China) was used to measure the extracellular conductivity (μS·cm^−1^).

The effect of SPA on lipid peroxidation was detected by measuring H_2_O_2_ and MDA contents. The hyphae (1 g, fresh weight) treated with SPA (0 and 1.0 mg/mL) were harvested for evaluation, as described earlier. Then, they were added to 0.1 M PBS buffer (pH 7.2) and ground in liquid nitrogen to homogenize the samples. The homogenate was centrifuged at 4°C for 10 min at 12,000 rpm, and the supernatants were collected for later use. The H_2_O_2_ and MDA contents were determined according to the manufacturer’s instructions.

### 2.5 Effect of SPA on mycelial morphology and ultrastructure

#### 2.5.1 Scanning electron microscopy

The morphological effect of SPA on *D. mahothocarpus* mycelial growth was investigated by standard scanning electron microscope (SEM). *Diaporthe mahothocarpus* was incubated in a 28°C incubator with built-in light bulbs (4,000 lx), and then, the hyphae (1–2 mm^2^) were separately obtained from the 3-day-old medium with SPA (0 and 1.0 mg/mL) and were treated using the method described by Ji et al. (2018) for observation under SEM (SU8100, HITACHI, Tokyo, Japan) at 4000× magnification.

#### 2.5.2 Transmission electron microscopy

The effect of SPA on *D. mahothocarpus* mycelial ultrastructure during growth was investigated by transmission electron microscope (TEM). Hyphae (1 g, fresh weight) were collected as described above, and sample preparation was performed according to the method by Chen et al. (2023). The hyphae were fixed in 2% (*v*/*v*) glutaraldehyde solution at room temperature for 4 h and then rinsed two to three times with 0.1 M phosphate buffered saline (PBS, pH 7.4) for 15 min each time. Then, they were fixed with 1% samarium tetroxide (OsO_4_) in 0.1 MPB (pH 7.4) for 2 h at 4°C and dehydrated with an ethanol gradient (30, 50, 70, 80, 95, and 100%), with the treatment time of each ethanol concentration being 20 min. After the samples were embedded for 24 h, the hyphal sections of approximately 70 nm in thickness were cut with an ultramicrotome (EM UC7, LEICA, Wetzlar, German) and stained with 2% uranyl acetate and 2.6% lead citrate. Finally, the samples were observed under TEM (HT7800, HITACHI) at 15,000× magnification.

### 2.6 Effect of SPA on oxidative stress of *Diaporthe mahothocarpus*

#### 2.6.1 Measurement of ROS

ROS production was measured using a 2′,7′-dichlorofluorescein diacetate (DCFH-DA) fluorescent assay and expressed as fluorescent intensity according to the method described by Kim and Xue (2020) with slight modifications. The spore suspension of *D. mahothocarpus* was added to 50 mL of PDB containing 1.0 mg/mL SPA. After being cultured at 28°C for 6 h, the cells were collected by centrifugation and washed twice with 0.1 M PBS (pH 7.2). The collected cells were incubated with 20 μM DCFH-DA solution for 40 min at 37°C in the dark and then centrifuged at 2,000 rpm for 5 min and washed thrice with 0.1 M PBS (pH 7.2). The sample was resuspended in 2 mL of 0.1 M PBS (pH 7.2) for the determination of fluorescent intensity with a fluorescence spectrophotometer (Cary Eclipse, Agilent Technologies, Santa Clara, CA, USA) at an excitation wavelength of 488 nm and an emission wavelength of 525 nm.

#### 2.6.2 Assessment of DNA damage

A comet assay was used to assess the effect of SPA on DNA damage in *D. mahothocarpus* (Olive and Banath, 2006). The spore suspension of *D. mahothocarpus* was added to 50 mL of PDB containing 1.0 mg/mL SPA. After being cultured at 28°C for 24 h, the cells were collected by centrifugation and washed twice with precooled 0.1 M PBS (pH 7.2). Then, they were centrifuged and resuspended in 0.1 M PBS (pH 7.2) to a density of 1 × 10^6^ cells/ml. Next, the prepared samples were successively sectioned and subjected to cell lysis, DNA de-rotation, and electrophoresis according to the instructions of the kit. After neutralization and staining, the samples were finally observed under a fluorescence microscope (M205FA, Leica) at an excitation wavelength of 535 nm and an emission wavelength of 617 nm, and 50 cells were randomly selected from each sample to measure the length of the tail of the damaged DNA. The tail DNA% and tail moment were calculated according to the following formulas:


$$\mathrm{Tail}\;DNA\;\left(\%\right)=\frac{\mathrm{Tail}\;DNA\;\mathrm{Intensity}}{\mathrm{Cell}\;DNA\;\mathrm{Intensity}}\times 100\text{,}$$

TailMoment=TailDNA×TailMomentLength.

The tail moment length was measured from the center of the head to the center of the tail.

#### 2.6.3 Measurement of reduced glutathione level

The spore suspensions of *D. mahothocarpus* were inoculated into PDB containing 1.0 mg/mL SPA and cultured at 160 rpm under light (4,000 lx). An equal volume of distilled water was added as a control. After incubation for 6 h, 12 h, 24 h, 48 h, and 72 h, the hyphae were collected by filtration and then centrifuged at 8,000 rpm for 10 min.

The homogenate was collected according to the method described in Section 2.4 and then centrifuged at 3500 rpm for 10 min. The supernatant was collected to measure the T-GSH/GSSG content (G0206W) using the Suzhou Grace Biotechnology commercial kits. The reduced glutathione (GSH) content was calculated as the T-GSH content minus twice the GSSG content.

#### 2.6.4 Determination of SOD activity and CAT activity

The hyphae were collected as described in Section 2.6.3, and the homogenate was collected according to the method mentioned in Section 2.4 and then centrifuged at 8000 rpm for 5 min. The supernatant was then collected to measure the SOD (G0101W) and CAT (G0105W) activity levels using Suzhou Grace Biotechnology commercial kits.

### 2.7 Effect of SPA on apoptosis and necrosis of *Diaporthe mahothocarpus*

The Annexin V-FITC/PI Co-stain can be used to detect apoptotic/necrotic cells (Wang et al., 2019). The spore suspension of *D. mahothocarpus* was added to 50 mL of PDB containing 1.0 mg/mL SPA. After being cultured at 28°C for 12 h, the cells were collected by centrifugation at 1,600 rpm for 5 min and washed twice with 0.1 M PBS (pH 7.2). Then, the cells were resuspended in 500 μL of binding buffer, after which 5 μL of Annexin V-FITC and 5 μL of PI were added separately. The samples were incubated at room temperature in the dark for 10 min. After incubation in the dark at room temperature for 10 min, the samples were immediately detected using a fluorescence microscope (M205FA, Leica).

### 2.8 Transcriptome sequencing

To analyze the response of *D. mahothocarpus* to SPA, the mycelia were collected as described in Section 2.4 and immediately frozen in liquid nitrogen. Total RNA was extracted using the TRIzol reagent (Thermo Fisher, Waltham, MA, USA, 15596018) following the manufacturer’s procedure. The total RNA quantity and purity were analyzed using a Bioanalyzer 2,100 and RNA 6000 Nano LabChip Kit (Agilent Technologies, 5,067–1,511), respectively, and high-quality RNA samples (RIN > 7.0) were used to construct a sequencing library. After total RNA was extracted, mRNA was purified from total RNA (5 μg) using Dynabeads Oligo (dT) (Thermo Fisher) with two rounds of purification. Following the purification process, mRNA was fragmented into short fragments using divalent cations at a high temperature [Magnesium RNA Fragmentation Module (NEB, Ipswich, MA, USA, cat.e6150)] of 94°C for 5–7 min. Then, the cleaved RNA fragments were reverse-transcribed to create cDNA using SuperScript^™^ II Reverse Transcriptase (Invitrogen, Carlsbad, CA, USA, cat. 1,896,649), which was next used to synthesize U-labeled second-stranded DNA with *E. coli* DNA polymerase I (NEB, cat.m0209), RNase H (NEB, cat.m0297), and dUTP Solution (Thermo Fisher, cat.R0133). An A-base was then added to the blunt ends of each strand, preparing them for ligation to the indexed adapters. Each adapter contained a T-base overhang for ligating the adapter to the A-tailed fragmented DNA. Dual-index adapters were ligated to the fragments, and size selection was performed with AMPureXP beads. After the heat-labile UDG enzyme (NEB, cat.m0280) treatment of the U-labeled second-stranded DNAs, the ligated products were amplified by PCR under the following conditions: initial denaturation at 95°C for 3 min, eight cycles of denaturation at 98°C for 15 s, annealing at 60°C for 15 s, and extension at 72°C for 30 s, and then a final extension at 72°C for 5 min. The average insert size for the final cDNA libraries was 300 ± 50 bp. Finally, 2 × 150 bp paired-end sequencing (PE150) was performed on Illumina Novaseq^™^ 6000 (Illumina, San Diego, CA, USA) at LC-Bio Technology Co., Ltd. (Hangzhou, China), following the manufacturer’s recommended protocol.

In brief, to obtain high-quality clean reads, reads were further filtered by Cutadapt^1^ (Martin, 2011). After removing the low-quality reads and adaptors, clean reads were mapped against the predicted transcripts of the *D. mahothocarpus* genome using HISAT2.^2^ The mapped reads of each sample were assembled using StringTie^3^ with default parameters. Then, all transcriptomes from all samples were merged to reconstruct a comprehensive transcriptome using GffCompare.^4^ After the final transcriptome was generated, StringTie and ballgown^5^ were used to estimate the expression levels of all transcripts and expression abundance for mRNAs by calculating the fragment per kilobase of transcript per million mapped reads (FPKM) value. Differential gene expression analysis was performed using DESeq2 software on comparisons between two different groups (and by using edgeR for comparisons between two samples). The genes with a false discovery rate (FDR) below 0.05 and an absolute fold change of at least 2 were considered differentially expressed genes (DEGs). The identified DEGs were then subjected to enrichment analysis of Gene Ontology (GO) terms and Kyoto Encyclopedia of Genes and Genomes (KEGG) pathways using Blast2GO (Bioinformatics Department, Valencia, Spain) and OmicShare.^6^

### 2.9 Quantitative real-time PCR (RT-qPCR) validation

To validate the reliability of the RNA sequencing results, RT-qPCR was conducted on 10 randomly selected DEGs that met a threshold of |log_2_(fold change)| > 5. Samples with the same treatment for RNA sequencing were used for cDNA synthesis, and the first-strand cDNA was synthesized according to the instructions of the kit (Sangon Biotech, Shanghai, China). The primers, as shown in Table 1, were designed using Primer 5 design software. For RT-qPCR, 2× SYBR Green PCR Master mix (Sangon Biotech, B110031) was used, and the procedure was performed using a CFX96 Real-Time System (Bio-Rad Laboratories, Shanghai, China), according to the following thermal cycling conditions: 95°C for 30 s, 40 cycles of 95°C for 10 s, and 60°C for 30 s. The levels of the target genes were compared with those of the housekeeping gene *α-actinin* using the 2^-ΔΔCt^ method (Yang et al., 2020). The means and standard errors were obtained from the average of three independent samples, and three biological replications of each treatment were performed.

**Table 1:** Primer sequences used for RT-qPCR analysis.

Gene ID	Gene name	Descriptions	Forward primer (5′-3′)	Reverse primer (5′-3′)	Anneal temperature	Product length
Dmahothocarpusptg000008lG013170	*DmRTA1*	Putative RTA1 like protein	GGGTTTCGTTTCTTTATC	CCAACTGGAAGTCTGCCT	50°C	232 bp
Dmahothocarpusptg000004lG021610	*DmMFS1*	Putative major facilitator superfamily transporter	TTTTGGGATTATGAACCG	GTGAGAATGAACTGGGAC	50°C	248 bp
Dmahothocarpusptg000004lG000690	*DmBG*	Beta-glucosidase	TACTGGGACTTCGAGAACG	GAGGTGGGCAAAGATAATG	52°C	249 bp
Dmahothocarpusptg000002lG006070	*DmFOXRED*	Putative fad dependent oxidoreductase superfamily	GTGCCTGCTTGTTCCCTA	GACGACTTTATCCGCTTT	52°C	180 bp
Dmahothocarpusptg000004lG007520	*DmPCMTD1*	Putative methyltransferase domain-containing protein	TCGGCACCTTCACGCACA	CGTCACCCCGCAACCCTT	60°C	212 bp
Dmahothocarpusptg000002lG006230	*DmTRI11*	Trichothecene c-15 hydroxylase	CGGGAGCAAGGTGAACAT	GCTGGAATCGGGAAGTAA	54°C	206 bp
Dmahothocarpusptg000005lG000240	*DmXEG1*	Putative glycoside hydrolase family 12 protein	ATTTGGAAGTGGGGCTA	GGCGACGAAGGAGTAGA	50°C	243 bp
Dmahothocarpusptg000002lG004330	*DmLIP1*	Putative lipase 1	GCAGGCCCGCGACTACT	CCGAGCCCCAGGGTGAA	60°C	219 bp
Dmahothocarpusptg000006lG020620	*DmPEL*	Putative pectate lyase	AGGGGGCTCGTCTGTGG	AAGGCGATGCTGTTGGT	54°C	205 bp
Dmahothocarpusptg000004lG025740	*DmCPR1*	Putative bifunctional P450/NADPH-P450 reductase	CACCTGACCCTGACCTACGC	TGGACGAAACCACGGAAA	58°C	230 bp
Dmahothocarpusptg000002lG015280	*DmACTN1*	*α-actinin*	CCGCCAAGCCAAAACTC	CACCACAGCAACAGACC	52°C	223 bp

### 2.10 Statistical analysis

Excel 2019 (Microsoft Corp., Redmon, WA, USA) was used to calculate mean values and standard deviations (*n* = 3). One-way ANOVA was performed using SPSS Statistics 24.0 (IBM Corp., Armonk, NY, USA). Statistical significance was evaluated at a threshold of *p* < 0.05. The level of DNA damage was measured using CASP 1.2.3 beta1 (CaspLab).^7^ Origin Pro 9.1 (OriginLab Inc., Northampton, MA, USA) and Publisher 2019 (Microsoft Corp.) were used to create all graphics.

## 3 Results

### 3.1 Effect of SPA on *Diaporthe mahothocarpus* mycelial growth and spore germination

**Figure 1:**
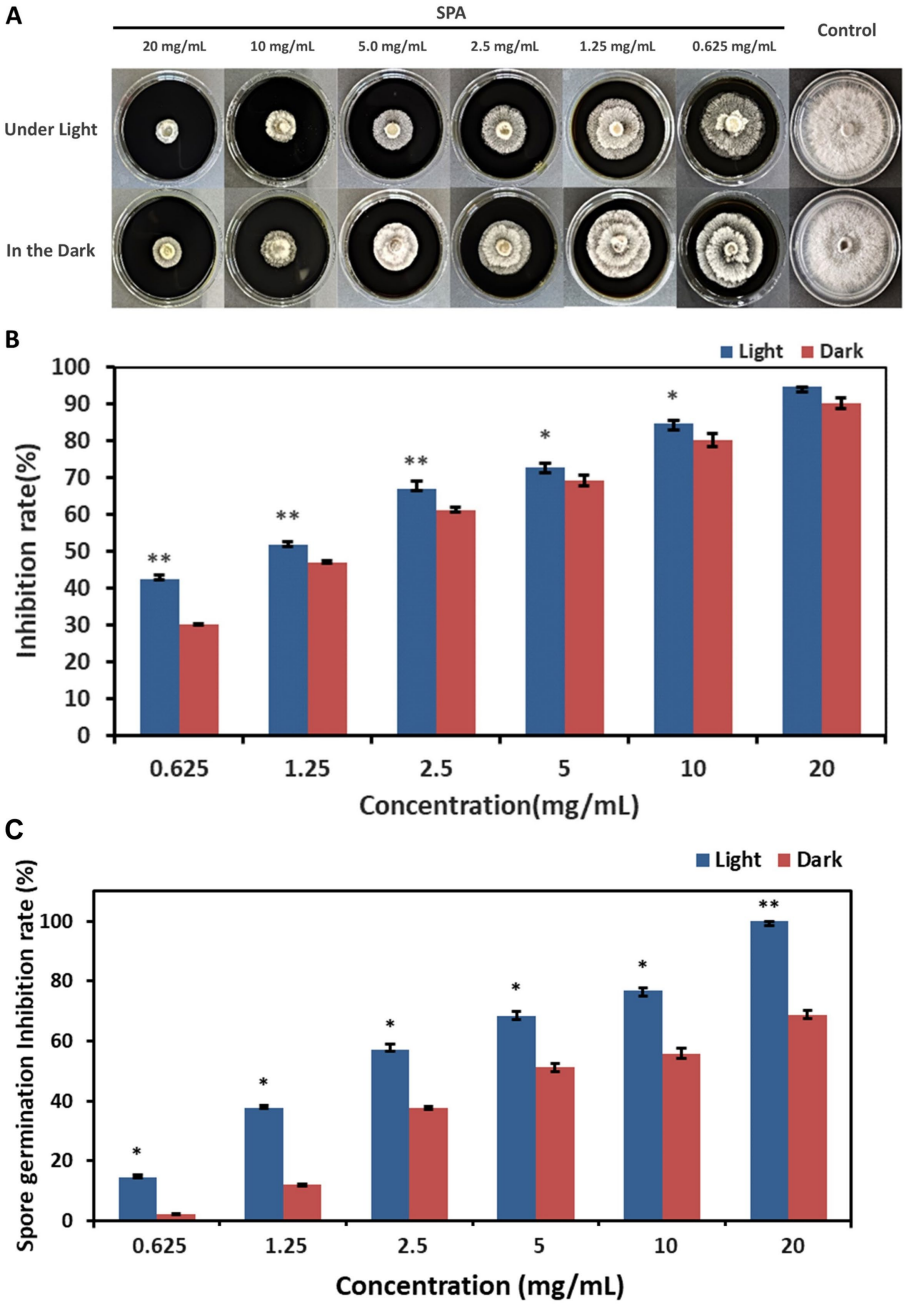
Effects of different concentrations of sodium pheophorbide a (SPA) on *Diaporthe mahothocarpus*. **(A,B)** The mycelial growth **(A)** and inhibition rate **(B)** of *D. mahothocarpus* treated with different SPA concentrations (0.625, 1.25, 2.5, 5, 10, and 20 mg/mL) were calculated after cultivation at 28°C for 5 days. **(C)** Spores were treated with different SPA concentrations (0.625, 1.25, 2.5, 5, 10, and 20 mg/mL), and the inhibition rate was calculated after incubation at 28°C for 24 h. The bars represent the standard error of the mean (*n* = 3), and asterisks indicate that the value under light is significantly different from that in the dark according to an independent samples *t*-test: *p < 0.05; **p < 0.01.

*Diaporthe mahothocarpus* grew normally without suppression in control conditions both under light and dark, whereas growth was inhibited in the SPA-treated groups (Figure 1A). SPA treatment significantly inhibited *D. mahothocarpus* mycelial growth (*p* < 0.05) in a concentration-dependent manner compared with the control (Figure 1B). When the concentration of SPA was 20 mg/mL, the highest inhibition rate was achieved, reaching 94.60% under light conditions and 90.16% in the dark. The presence or absence of light had a significant effect on its antifungal activity. Probit analysis with SPSS was used to determine the EC_50_ values and the virulence regression equations of SPA against *D. mahothocarpus* under light and dark conditions (Table 2).

**Table 2:** Toxicity of SPA against *D. mahothocarpus* under light (4,000 lx) and dark conditions.

	Culture condition	Toxicity equation	*χ^2^*	Correlation coefficient (R^2^)	EC_50_ (mg/mL)	95% Confidence interval (mg/mL)	EC_90_ (mg/mL)	95% Confidence interval (mg/mL)
Mycelial growth inhibition	Light	*Y* = 1.095X-0.027	2.477	0.992	**1.059**	**0.738–1.392**	15.690	10.784–26.919
	Dark	*Y* = 1.136X-0.234	0.992	0.9848	1.606	1.211–2.027	21.584	14.669–37.330
Spore germination inhibition	Light	*Y* = 1.681X-0.604	15.672	0.948	**2.287**	**1.314–3.589**	13.231	7.315–47.009
	Dark	*Y* = 1.406X-1.169	15.336	0.909	6.786	4.072–14.285	55.371	22.283–590.288

The bold values are EC50 values and confidence intervals of SPA inhibiting *D. mahothocarpus* mycelial growth and spore germination under light conditions.

Different SPA concentrations also had significant inhibitory effects (*p* < 0.05) on *D. mahothocarpus* spore germination under light conditions in a concentration-dependent manner (Figure 1C). Significant differences in the ability of SPA to inhibit spore germination were observed under light and dark conditions (*p* < 0.05). The inhibitory effect of 20.0 mg/mL SPA on spore germination under light conditions was 100% but only 68.84% under dark conditions. The EC_50_ values and the virulence regression equations of SPA against *D. mahothocarpus* under light and dark conditions are also shown in Table 2.

Moreover, the protective and control effects of SPA are shown in Table 3. Generally, the disease incidence of *C. oleifera* seedlings treated with SPA was significantly decreased relative to the control plants, and the protective effect was stronger than the control effect. When the concentration of SPA was 20 mg/mL, disease incidence in the prevention treatment group was only 3.52%, with the control efficiency reaching as high as 91.35%, showing strong *in vivo* antifungal effects.

**Table 3:** The *in vivo* inhibitory effect of SPA on *D. mahothocarpus*.

	Concentration of SPA (mg/mL)	Disease incidence	Control efficiency (%)
	0 (Control)	43.36 ± 3.14	\
Protective effect	10	5.05 ± 2.94	88.35 ± 6.79
	20	3.52 ± 0.23	91.3 ± 8.65
Control effect	10	14.20 ± 3.25	67.24 ± 7.51
	20	6.32 ± 2.38	85.43 ± 5.50

### 3.2 Effect of SPA on cell membrane permeability and lipid peroxidation

**Figure 2:**
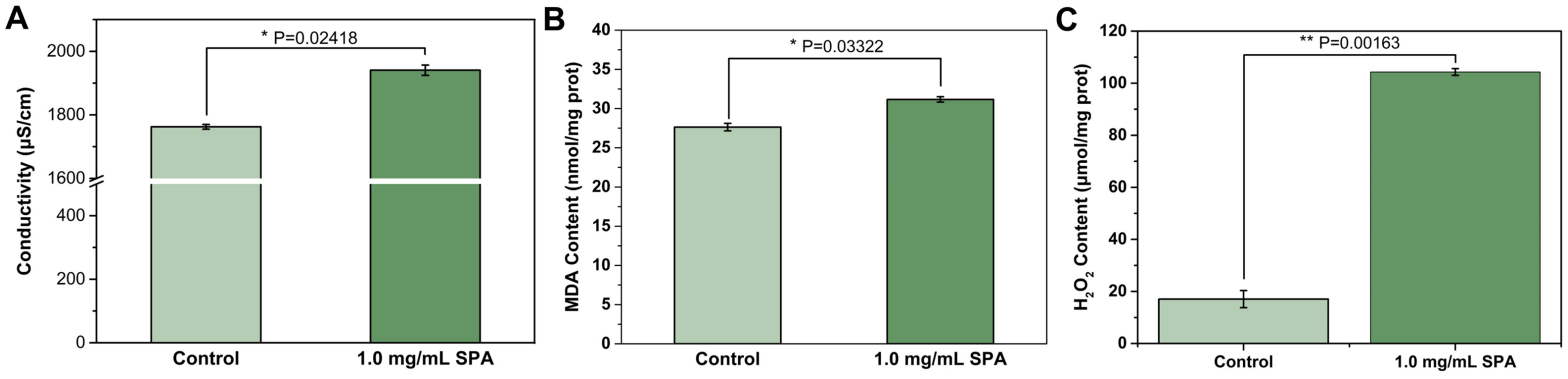
Conductivity **(A)**, malondialdehyde (MDA) content **(B)**, and H_2_O_2_ content **(C)** of *Diaporthe mahothocarpus* treated with 1.0 mg/mL SPA under light (4,000 lx) conditions. Values are means and standard errors (*n* = 3), and asterisks indicate that a value is significantly different from the control according to an independent samples *t*-test.

The extracellular conductivity was measured to evaluate cell membrane permeability. The conductivity of *D. mahothocarpus* (Figure 2A) treated with 1.0 mg/mL SPA was significantly higher than that of the control (*p* < 0.05), increasing by approximately 10.11%.

MDA and H_2_O_2_ are important indexes for the evaluation of the degree of membrane peroxidation. As shown in Figure 2B, SPA increased MDA content in *D. mahothocarpus*. After 1.0 mg/mL of SPA treatment, the MDA content in *D. mahothocarpus* hyphae reached 31.16 nmol/mg protein, an increase in 12.78% relative to that of the control. Similarly, the H_2_O_2_ content in *D. mahothocarpus* hyphae significantly increased (*p* < 0.05) to 6.11 times that of the control (Figure 2C).

### 3.3 Effect of SPA on mycelial morphology and ultrastructure

**Figure 3:**
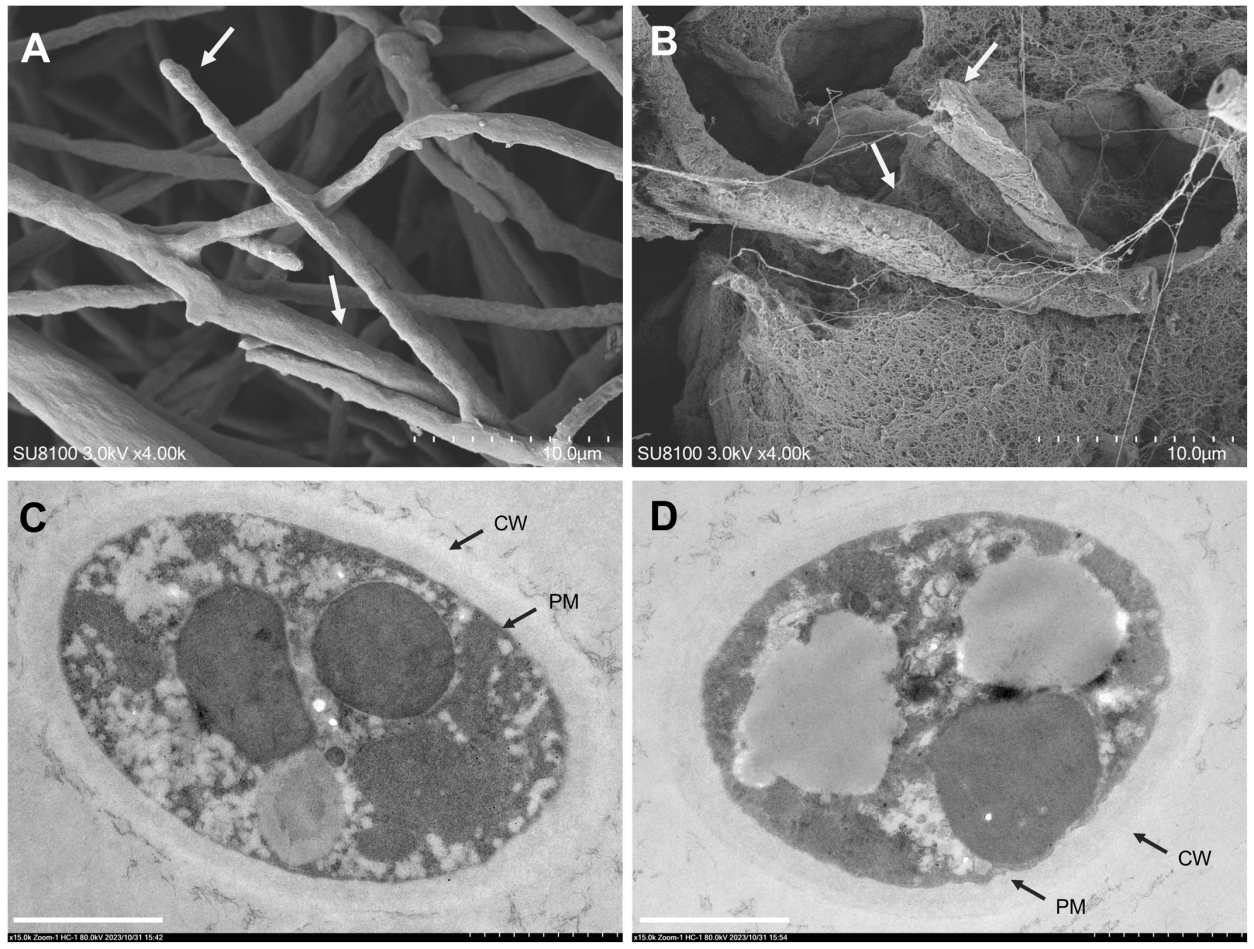
Effects of sodium pheophorbide a (SPA) on *Diaporthe mahothocarpus* mycelial morphology and ultrastructure after treatment. **(A,B)** Scanning electron microscopy images of *D. mahothocarpus* treated with 0 **(A)** and 1.0 mg/mL SPA **(B)** under 4,000× magnification; the dotted scale with 10 small grids is 10.0 μm. **(C,D)** Transmission electron microscopy images of *D. mahothocarpus* treated with 0 **(A)** and 1.0 mg/mL of SPA **(B)** under 80,000× magnification; CW, cell wall; PM, plasma membrane; scale bar, 1.0 μm **(B)**.

SEM was used to determine the effects of SPA (0 and 1.0 mg/mL) on the surface morphology of *D. mahothocarpus* mycelia (Figures 3A,B). The untreated hyphae were regularly distributed and had intact morphological structures. Their surfaces were smooth and full, with round hyphal tips (Figure 3A). However, the mycelia showed abnormalities when exposed to SPA; they were rough, intertwined, swollen, and even fractured after 1.0 mg/mL SPA treatment (Figure 3B).

As shown in Figure 3C, untreated *D. mahothocarpus* hyphae exhibited normal cytological and ultrastructural characteristics of nutrient hyphae, with uniform cell walls and cell membrane structures, rich cytoplasmic matrixes, and intact organelles. In contrast, extensive cell wall thickness, detached cytoplasmic membranes, and degradation of the cytoplasmic matrix and organelles were observed in *D. mahothocarpus* hyphae treated with 1.0 mg/mL SPA (Figure 3D).

### 3.4 Effect of SPA on oxidative stress in *Diaporthe mahothocarpus*

#### 3.4.1 ROS accumulation

**Figure 4:**
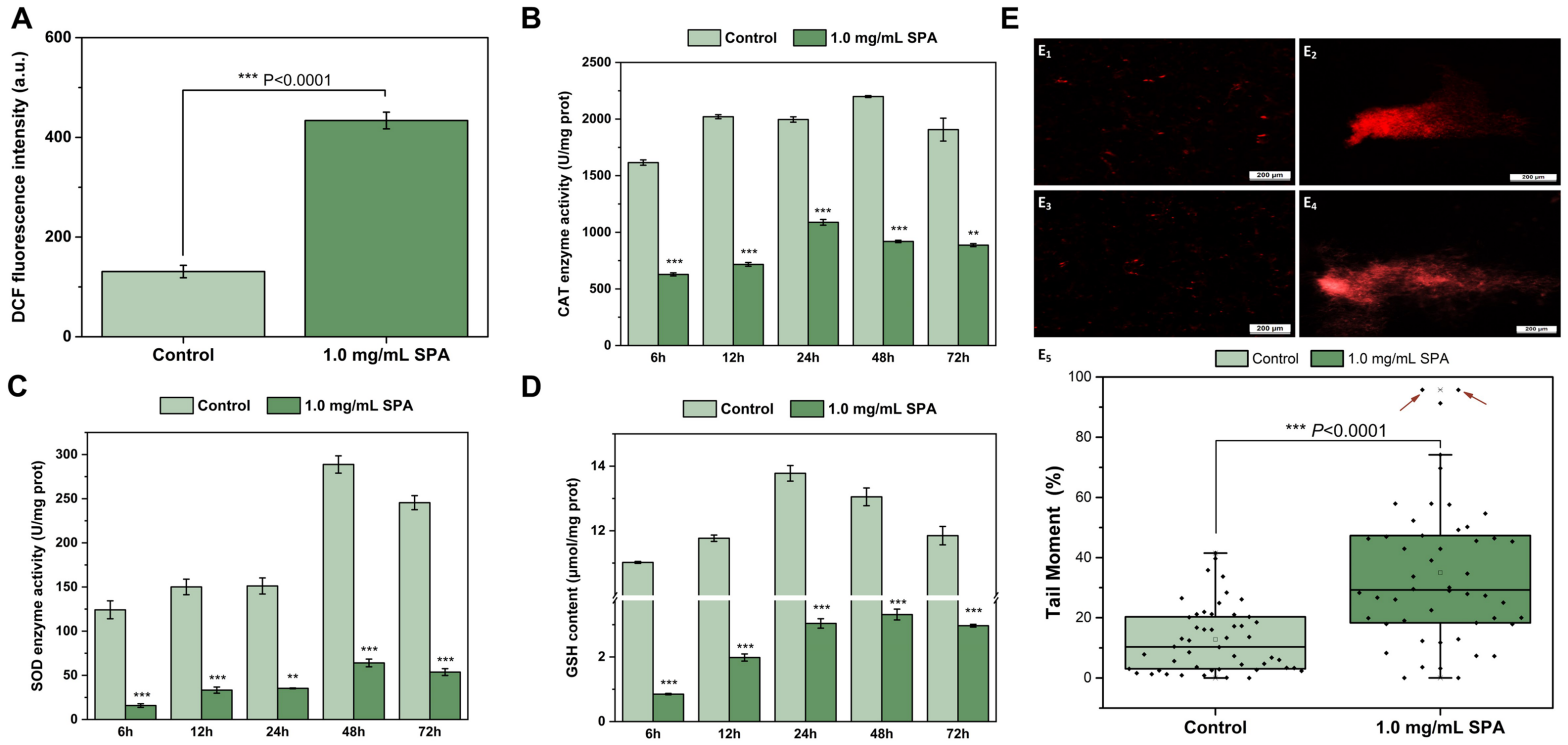
Effects of sodium pheophorbide a (SPA) on oxidative stress of *Diaporthe mahothocarpus*. **(A–D)** Effects of SPA on reactive oxygen species (ROS) accumulation **(A)**, enzyme activities of catalase (CAT) **(B)** and superoxide dismutase (SOD) **(C)** and glutathione (GSH) content **(D)** of *D. mahothocarpus*. *D. mahothocarpus* was treated with different SPA concentrations (0 mg/mL, as control, and 1.0 mg/mL). The bars represent the standard error of the mean (*n* = 3). Effects of SPA on DNA damage. **(E**_**1–4**_**)** Comet images of untreated **(E**_**1,2**_**)** and SPA-treated **(E**_**3,4**_**)** spores of *D. mahothocarpus*. **(E**_**5**_**)** Tail moments of 50 randomly selected untreated **(E**_**1,2**_**)** and SPA-treated **(E**_**3,4**_**)** spores of *D. mahothocarpus*. The red arrows point to two severely damaged spores (tail moment >95%).

The effects of SPA at concentrations of 0 and 1.0 mg/mL on ROS accumulation in *D. mahothocarpus* are shown in Figure 4A; 1.0 mg/mL SPA significantly induced *D. mahothocarpus* to produce a large amount of ROS (*p* < 0.05), with the fluorescence intensity of DCF 3.32 times higher than that of the control.

#### 3.4.2 CAT and SOD enzyme activities and GSH level

The effects of SPA at concentrations of 0 and 1.0 mg/mL on CAT and SOD enzyme activities and GSH level in *D. mahothocarpus* are shown in Figures 4B–D. Compared with the control, CAT (Figure 4B) and SOD (Figure 4C) enzyme activities and the GSH level (Figure 4D) in the SPA-treated group showed the same change trend with the increase in incubation time, but they were significantly decreased. It can be observed from Figure 4B, SPA significantly decreased the enzyme activities of CAT, which were 2.57, 2.82, 1.83, 2.39, and 2.15 times lower than those of the control, respectively. Notably, the enzyme activities of SOD in *D. mahothocarpus* increased sharply at 48 h and decreased slightly at 72 h, while they were 4.51 and 4.57 times lower in SPA-treated mycelia than those in the control, respectively (Figure 4C). Additionally, the GSH level in the control first increased and then decreased and returned to the level of 6 h at 72 h, while the GSH levels in *D. mahothocarpus* treated with SPA were significantly reduced at only 7.71–25.03% that of the control group (Figure 4D).

#### 3.4.3 DNA damage

A comet assay was conducted to evaluate the effect of SPA on DNA damage, and the comet images are shown in Figures 4E_1–4_. Fifty *D. mahothocarpus* spores were randomly selected from each treatment group, and the tail moment was calculated using the above formula. Treatment with 1.0 mg/mL of SPA resulted in significantly higher tail moments in the comet assay compared with the control (Figure 4E_5_). Approximately 68% of spores had at least moderate DNA damage, while over 70% of spores in the control had DNA damage levels below 20%.

### 3.5 Effect of SPA on cell death in *Diaporthe mahothocarpus*

**Figure 5:**
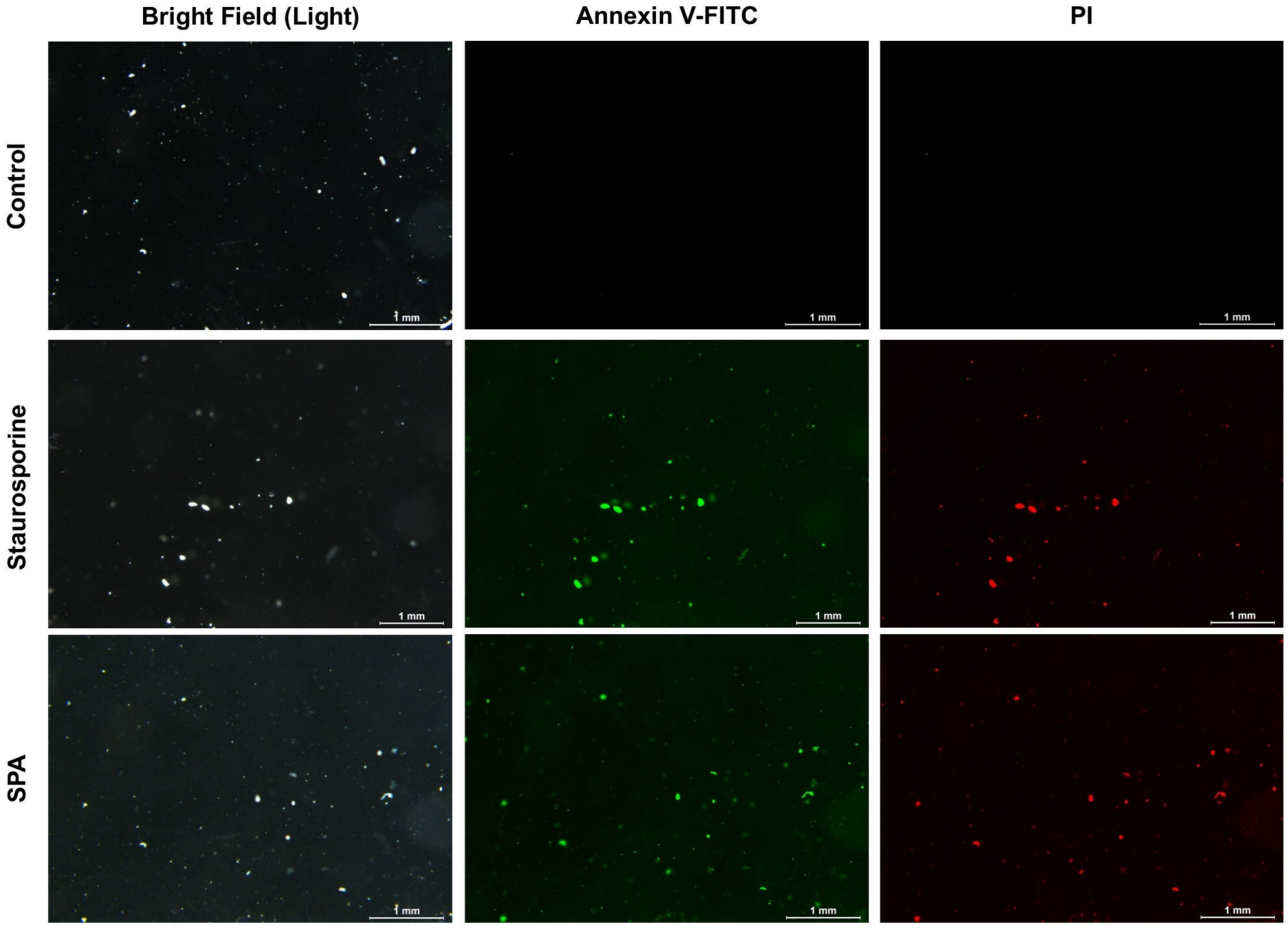
Annexin V-FITC and PI staining of *Diaporthe mahothocarpus* spores after treatment with 1.0 mg/mL sodium pheophorbide a (SPA). Staurosporine was used as a positive control; scale bar = 1.0 mm.

As shown in Figure 5, spores in the control group were not stained by Annexin V-FITC or PI, indicating that they were living cells. However, spores treated with 1.0 mg/mL SPA were annexin- and PI-positive, suggesting that the later stage of the apoptosis process had already conducted and most cells were dead.

### 3.6 Transcriptomic analysis of *Diaporthe mahothocarpus* with SPA treatment

#### 3.6.1 Analysis of DEGs

**Figure 6:**
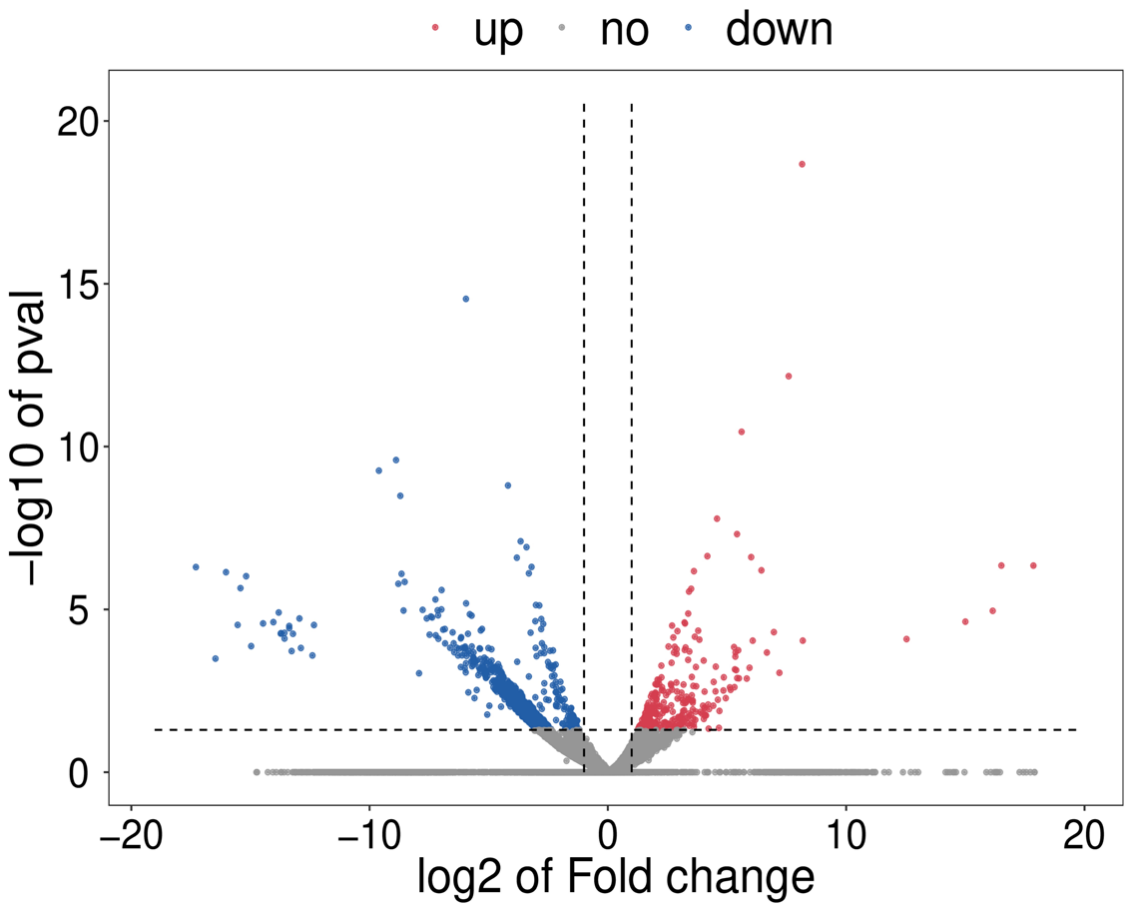
Volcano plot. For each plotted gene, the abscissa shows log_2_ (fold change), and the ordinate is -log_10_ (*p*-value). The red dots represent significantly upregulated differentially expressed genes (DEGs), blue dots represent significantly downregulated DEGs, and gray dots represent genes with no significant difference in expression.

To further reveal the effect of SPA on mycelial growth and metabolism of *D. mahothocarpus*, comparative transcriptome analysis was conducted. Compared with the untreated control group, 1,105 genes were differentially expressed in *D. mahothocarpus* treated with 1.0 mg/mL of SPA. Among these DEGs, 296 were upregulated and 809 were downregulated (Figure 6). The number of downregulated genes was more than 2.7 times that of upregulated genes, which indicated that SPA inhibited the expression of genes in *D. mahothocarpus*.

#### 3.6.2 Functional annotation and enrichment analysis of DEGs

**Figure 7:**
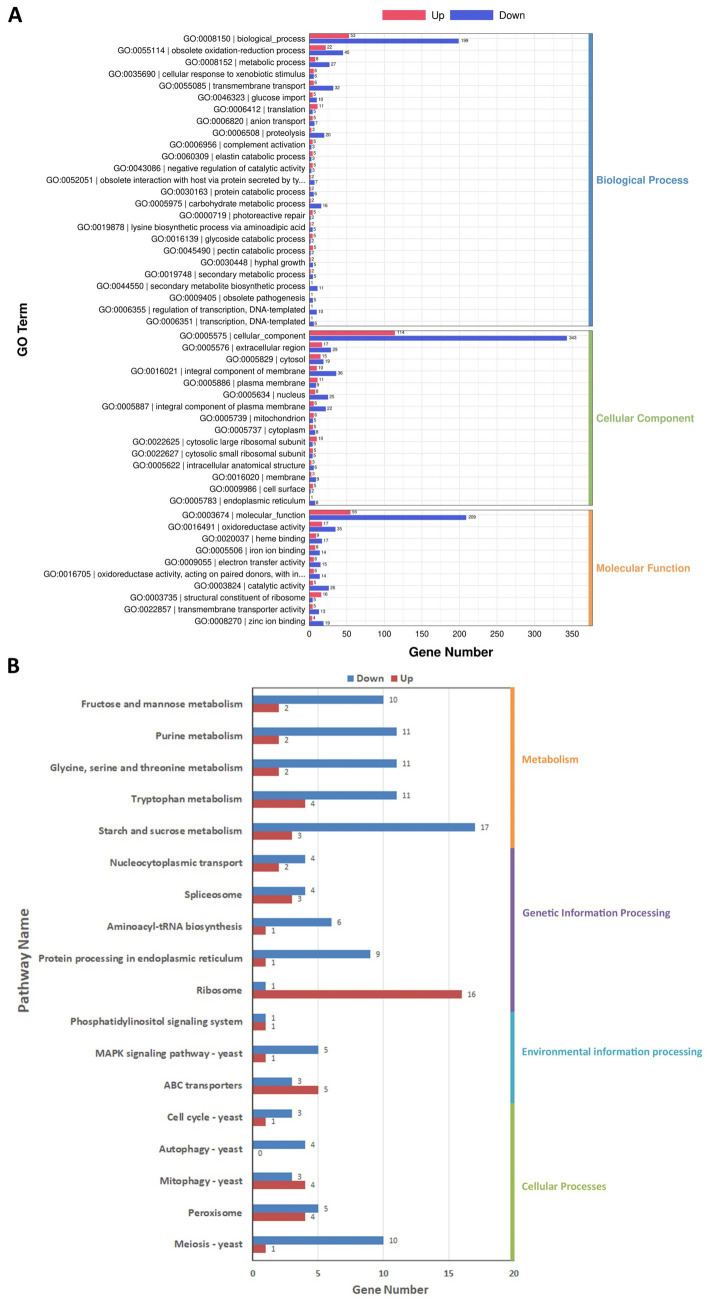
Gene Ontology (GO) **(A)** and Kyoto Encyclopedia of Genes and Genomes (KEGG) **(B)** enrichment analysis of the differentially expressed genes (DEGs) in *Diaporthe mahothocarpus*. The abscissa represents the number of DEGs enriched in the various GO entries **(A)** or KEGG pathways **(B)**. Along the ordinate are shown the GO entries **(A)** or KEGG pathway names **(B)**. The number of upregulated genes is indicated by redness, and the downregulated genes is indicated by blueness.

DEGs were classified according to three categorizations, i.e., biological process, cellular component, and molecular function, in the GO enrichment analysis (Figure 7A). Among biological process ontology terms, the downregulated DEGs were mainly enriched for involvement in obsolete oxidation–reduction process (GO:0055114), transmembrane transport (GO:0055085), and metabolic process (GO:0008152). In the cellular component and molecular function ontologies, the dominant GO terms were integral component of membrane (GO:0016021) and oxidoreductase activity (GO:0016491).

Furthermore, DEGs were related to 98 pathways in the KEGG database. They were classified into four categories: cellular processes, environmental information processing, genetic information processing, and metabolism. As shown in Figure 7B, the downregulated DEGs were mainly involved in metabolism and cellular processes, with 17 and 10 downregulated DEGs in the starch and sucrose metabolism (ko00500) and meiosis-yeast (ko04113) pathways, respectively. Notably, there were 16 upregulated DEGs in the ribosome (ko03010) pathway, indicating that SPA might enhance the synthesis of ribosomal proteins in *D. mahothocarpus* mycelia.

#### 3.6.3 Validation of RNA-seq results by RT-qPCR analysis

**Figure 8:**
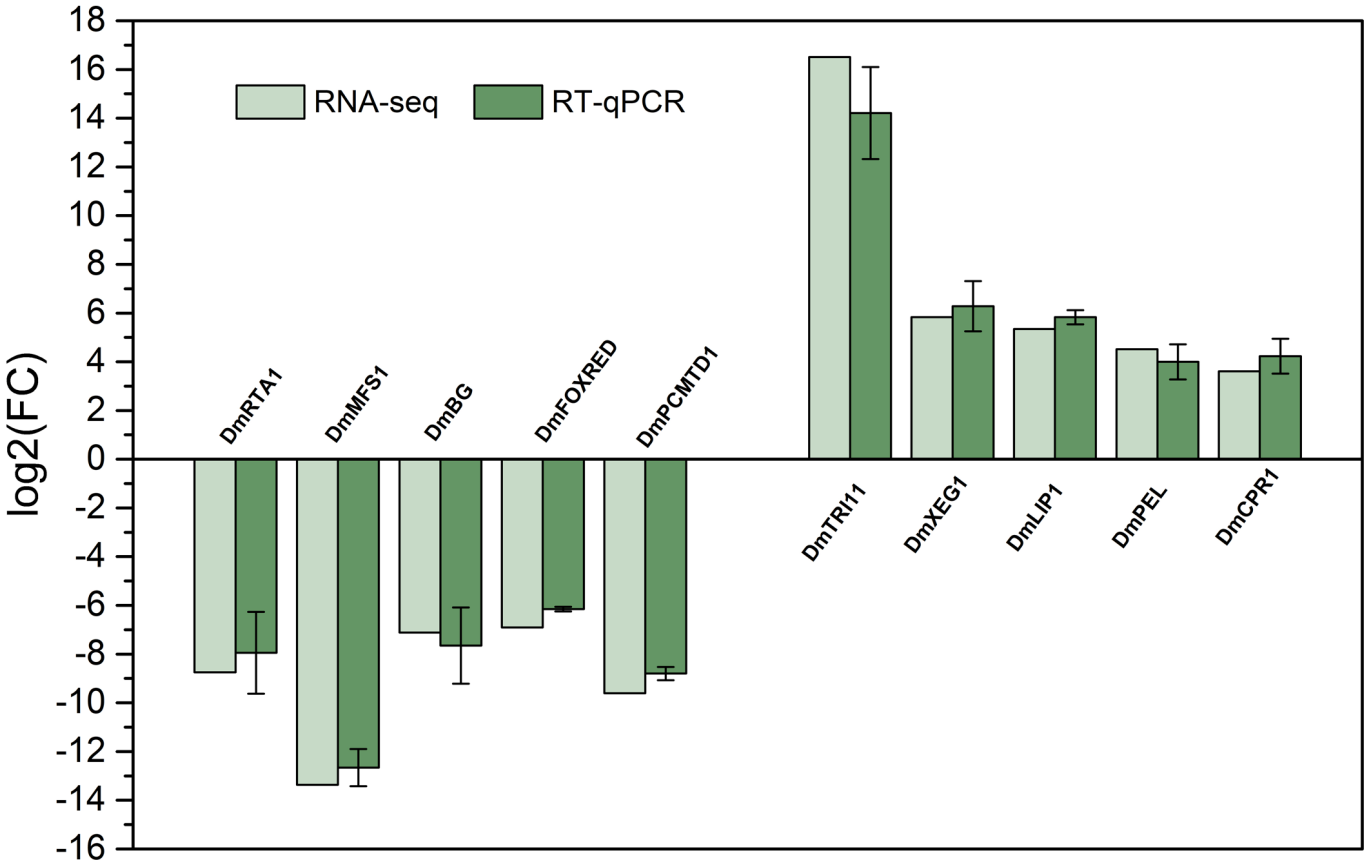
Comparison of the RNA-seq results and RT-qPCR results to validate the expression of 10 selected differentially expressed genes. Values of RT-qPCR are means and standard errors (*n* = 3).

To validate the DEGs identified by the RNA-seq results, 10 genes (containing 5 upregulated and 5 downregulated genes) were randomly selected from the results of RT-qPCR analysis. The 10 pairs of primers were designed (Table 1), and *α-actinin* (which did not significantly differ in gene expression) was used as an internal control for expression normalization. As shown in Figure 8, the expression of the selected genes was consistent with the RNA-Seq results, which indicated the reliability of the transcriptome sequencing.

## 4 Discussion

Photosensitizers are organic compounds that can produce ROS in the presence of light and have gradually gained the attention of agriculture and forestry researchers owing to their effectiveness in inhibiting microorganisms (Rodoni and Lemoine, 2023). Several compounds can act as photosensitizers, such as curcumin, chlorophyllins, hypericin, porphyrins, and coumarins. SPA, a type of porphyrin compound, is a natural photosensitizer with great potential for development as an environmentally friendly photoactivated fungicide (Nagai et al., 2014). However, its photoactivated antifungal activity against *D. mahothocarpus* has been unknown. Therefore, in the present study, the photoactivated antifungal activity and mode of action of SPA against *D. mahothocarpus* and its response were explored based on transcriptome sequencing.

Previous studies revealed that SPA has strong inhibitory effects on mycelial growth of several phytopathogens, and the EC_50_ values of SPA under light conditions on *P. neglecta* and *B. cinerea* were 8.574 and 9.770 mg/mL, respectively (Yang et al., 2019; Ji et al., 2020). In the present study, the EC_50_ value of SPA on *D. mahothocarpus* under the same light conditions was only 1.059 mg/mL, indicating that *D. mahothocarpus* is much more sensitive to SPA. Notably, similar to the research conducted by Krüger et al. (2019), who showed that *E. coli* NR698 and *Bacillus subtilis* could be inactivated by chlorophyllin in darkness (Krüger et al., 2019), SPA also showed substantial inhibitory effects in darkness, indicating a second, light-independent mode of action. Similarly, SPA has a much stronger inhibitory effect on spore germination of *D. mahothocarpus* than on spore germination of *P. neglecta* and *B. cinerea*. Notably, we found that SPA had a lower EC_50_ value for mycelial growth (1.059 mg/mL) compared to spore germination (2.287 mg/mL) in *D. mahothocarpus*. This suggests that for the future management of *C. oleifera* leaf spot blight, SPA may be more effective for control than protection. However, we have confirmed that the protective effect of SPA at both 10 and 20 mg/mL is still stronger than the control effect through *in vivo* experiments with potted seedlings, which may be owing to the concentrations used being closer to the estimated EC_90_.

Cell membranes form a fundamental part of all living cells (Kumar Yadav et al., 2019) and participate in various physiological processes (Mychack and Janakiraman, 2021). For filamentous pathogenic fungi, the cell membrane is not only a protective barrier, as its homeostasis can also directly and indirectly affect fungal pathogenicity (Peng and Chen, 2024). In our study, the transcriptome data suggested that many cell membrane-related genes were affected by SPA, including genes related to transport activity and integral membrane components. In addition to the ABC transporter, the major facilitator superfamily (MFS) transporters also play an important role in the transport processes. Membrane potential and electrochemical proton gradient drive the transport of compounds through membranes (Liu et al., 2017). Transcriptome data showed that 19 genes of MFS transporters were significantly downregulated (Supplementary Table 1), and RT-qPCR validated that SPA downregulated the gene expression level of *DmMFS1* encoded by Dmahothocarpusptg000004lG021610. Wang et al. (2012) revealed that the lack of *PdMfs1* would increase the sensitivity to imazalil by affecting the membrane transport function and reducing the virulence of *Penicillium digitatum* on citrus fruits. Moreover, a fungal lipid-translocating exporter family gene *DmRTA1*, which could work in multidrug resistance and membrane phospholipid homeostasis (Manente and Ghislain, 2009), was also significantly downregulated. These data are consistent with the SEM and TEM images, the increase in electrolyte leakage, and PI staining. Thus, we consider that SPA downregulated most MFS transporter genes and other transporting-related genes, ultimately leading to the reduction in cell membrane permeability, the inhibition of transmembrane transport, and the interference of intracellular substance transport function.

Many natural antifungal compounds can induce the accumulation of ROS in fungi, which may disrupt the imbalance of cellular redox homeostasis and ultimately lead to fungal cell death. For instance, Huo et al. (2023) reported that burst of ROS levels induced by *p*-hydroxybenzoic acid in *Aspergillus flavus* cells could lead to cell death (Huo et al., 2023). Notably, ROS is also an important factor in the effectiveness of photosensitizers. During the process of photoreaction, ROS produced by photosensitizers can act as fungicidal factors that attack microorganisms and damage fungal cells through the oxidation of essential molecular components such as proteins, lipids, and nucleic acids (Vatansever et al., 2013; Weijer et al., 2015), thereby inhibiting their growth (Leem et al., 2018). Transcriptome sequencing analysis revealed the molecular mechanism of SPA inhibiting *D. mahothocarpus* growth by inducing the oxidative homeostasis imbalance. DEGs with clear function annotation (not including hypothetical proteins) related to exogenous oxidative stress are shown in Supplementary Table 2, showing that SPA treatment significantly affected the intracellular oxidative stress level of *D. mahothocarpus*. Transcriptomic data indicated that the enzymes related to glutathione metabolism were significantly upregulated in SPA-treated *D. mahothocarpus* hyphae. A previous study reported that GSH metabolism in *Aspergillus flavus* played a key role in response to perillaldehyde stress, which induced an apoptosis process by accumulating ROS (Pan et al., 2020). GSH is the most abundant intracellular low molecular weight mercaptan with a variety of physiological functions, which is essential to protect ROS and signal molecules involved in cell proliferation, cell cycle regulation, and apoptosis (Presnell et al., 2013). In our study, we confirmed that the GSH content was decreased by SPA, indicating that GSH depletion may cause subsequent DNA damage, which was consistent with the observation in the comet assay. Furthermore, the excessive ROS was induced by SPA treatment (Figure 4A), with the decreased activities of SOD and CAT (Figures 4B–D) and increased MDA and H_2_O_2_ content, indicating that exposure of *D. mahothocarpus* to SPA induced a burst of ROS in spore cells and disrupted the imbalance of cellular redox homeostasis, thereby promoting cell apoptosis and necrosis. This result is consistent with the reported effect of curcumin, another photosensitizer, on *D. phaseolorum* (Kai et al., 2020). Interestingly, SPA induced upregulated expression of genes related to the ribosome, nucleic acid metabolism, and membrane protein synthesis-related biological process in *D. mahothocarpus*, which was speculated to be the positive regulation of *D. mahothocarpus* in response to external stress under SPA treatment. Therefore, we hypothesize that there are two main reasons for the massive increase in the intracellular ROS content after SPA treatment. On the one hand, SPA can produce a large amount of ROS itself when induced by light. On the other hand, SPA also inhibits the gene expression and enzyme activities of SOD, CAT, and other antioxidant-related enzymes, thereby interrupting the standard ROS clearance mechanisms, disrupting cellular redox homeostasis, inhibiting the normal growth of hyphae, and causing cellular oxidative damage until death.

However, in this study, we have not yet conducted an in-depth exploration of mitochondria, which play a central role in fungal apoptosis, and mitochondrial dysfunction is always associated with apoptosis (Bock and Tait, 2020). Previous studies have shown that SPA can cause dysfunction of *P. neglecta* mitochondria to some extent, downregulating the gene and protein expression of enzymes related to energy metabolism (Yang et al., 2020; Zhang et al., 2024). Thus, in subsequent study, the role of mitochondria in response to the photoactivated antifungal activity of SPA will be a focus of our research.

## 5 Conclusion

The present study evaluated the photoactivated activity of SPA on mycelial growth and spore germination of *D. mahothocarpus* and further clarified the mode of action of SPA based on transcriptomic data. SPA was able to induce ROS burst, promote oxidative stress and apoptosis, destroy the cell membrane and mycelial morphology and structure, and ultimately inhibit mycelial growth and spore germination. All these findings indicate that SPA is a promising plant-derived photoactivated fungicidal substance that can be used as an effective and safe tool for the integrated management of *Camellia oleifera* leaf spot blight and also as a potential lead compound for the development of new fungicides.

## Data availability statement

The raw sequencing reads have been deposited under NCBI BioProject accession number PRJNA1112549.

## Author contributions

X-LS: Formal analysis, Investigation, Writing – original draft. JY: Formal analysis, Funding acquisition, Supervision, Writing – review & editing. YZ: Investigation, Validation, Writing – review & editing. PQ: Investigation, Validation, Writing – review & editing. H-YZ: Methodology, Writing – review & editing. Y-ZC: Resources, Writing – review & editing.

## Footnotes

^1^https://cutadapt.readthedocs.io/en/stable/, version cutadapt-1.9.

^2^https://daehwankimlab.github.io/hisat2/, version hisat2-2.2.1.

^3^http://ccb.jhu.edu/software/stringtie/, version stringtie-2.1.6.

^4^http://ccb.jhu.edu/software/stringtie/gffcompare.shtml, version gffcompare-0.9.8.

^5^http://www.bioconductor.org/packages/release/bioc/html/ballgown.html

^6^www.omicshare.com/tools

^7^https://sourceforge.net/projects/casp/
