# Supplementary material for: The photoactivated antifungal activity and possible mode of action of sodium pheophorbide a on Diaporthe mahothocarpus causing leaf spot blight in Camellia oleifera
Source: Front Microbiol. 2024 Jun 13;15:1403478. doi: 10.3389/fmicb.2024.1403478 (PMC11208333; doi:10.3389/fmicb.2024.1403478)
Supplement: Supplementary file 1 [file Table_1.DOCX]

**Supplementary Table 1. DEGs related to major facilitator superfamily (MFS) transporter**

| Gene ID | Descriptions | log_2_FC | *P* value | Regulation |
| --- | --- | --- | --- | --- |
| Dmahothocarpusptg000001lG004770 | putative major facilitator superfamily transporter | -5.76 | 0.000 | down |
| Dmahothocarpusptg000001lG005580 | putative mfs transporter | -3.23 | 0.031 | down |
| Dmahothocarpusptg000001lG015110 | MFS general substrate transporter | -1.68 | 0.019 | down |
| Dmahothocarpusptg000002lG001670 | major facilitator superfamily transporter | -2.98 | 0.019 | down |
| Dmahothocarpusptg000002lG006090 | putative mfs drug efflux | -3.30 | 0.011 | down |
| Dmahothocarpusptg000002lG013600 | major facilitator superfamily transporter | -3.06 | 0.020 | down |
| Dmahothocarpusptg000003lG000030 | major facilitator superfamily transporter | -5.23 | 0.001 | down |
| Dmahothocarpusptg000003lG000410 | sugar porter family MFS transporter | -3.75 | 0.009 | down |
| Dmahothocarpusptg000003lG001950 | major facilitator superfamily transporter | -5.13 | 0.001 | down |
| Dmahothocarpusptg000003lG003940 | putative mfs quinate transporter | -3.79 | 0.004 | down |
| Dmahothocarpusptg000003lG005960 | major facilitator superfamily transporter | -3.48 | 0.007 | down |
| Dmahothocarpusptg000003lG017740 | putative mfs transporter | -3.30 | 0.012 | down |
| Dmahothocarpusptg000004lG005350 | putative mfs maltose | -2.80 | 0.024 | down |
| Dmahothocarpusptg000004lG012240 | putative mfs allantoate | -3.02 | 0.026 | down |
| Dmahothocarpusptg000004lG021610 | putative major facilitator superfamily transporter | -13.37 | 0.000 | down |
| Dmahothocarpusptg000004lG028930 | putative mfs sugar | -3.28 | 0.016 | down |
| Dmahothocarpusptg000005lG011420 | major facilitator superfamily transporter | -3.99 | 0.006 | down |
| Dmahothocarpusptg000008lG013890 | MFS multidrug transporter | -3.36 | 0.026 | down |
| Dmahothocarpusptg000009lG003310 | putative mfs multidrug transporter | -2.73 | 0.025 | down |
