# Supplementary material for: The photoactivated antifungal activity and possible mode of action of sodium pheophorbide a on Diaporthe mahothocarpus causing leaf spot blight in Camellia oleifera
Source: Front Microbiol. 2024 Jun 13;15:1403478. doi: 10.3389/fmicb.2024.1403478 (PMC11208333; doi:10.3389/fmicb.2024.1403478)
Supplement: Supplementary file 2 [file Table_2.DOCX]

**Supplementary Table 2. DEGs with clear annotations related to exogenous oxidative stress**

| Gene type in response to oxidative stress | Gene ID | Descriptions | log_2_FC | *P* value | Regulation |
| --- | --- | --- | --- | --- | --- |
| Zinc finger transcription factor | Dmahothocarpusptg000006lG001080 | zinc finger transcription factor | -2.74 | 0.03 | down |
|  | Dmahothocarpusptg000002lG018630 | putative zinc finger protein | -4.18 | 0.00 | down |
| The enzymes related to recombination and repair | Dmahothocarpusptg000005lG002810 | putative sir2-like protein | 2.05 | 0.01 | up |
|  | Dmahothocarpusptg000002lG007720 | putative 40s ribosomal protein s3 | 1.41 | 0.04 | up |
|  | Dmahothocarpusptg000004lG015150 | cryptochrome DASH | -3.80 | 0.00 | down |
|  | Dmahothocarpusptg000001lG010520 | MutS domain V | -2.17 | 0.01 | down |
| The enzymes related to glutathione metabolism | Dmahothocarpusptg000002lG008350 | putative glutathione s-transferase | 2.77 | 0.00 | up |
|  | Dmahothocarpusptg000003lG007650 | isocitrate dehydrogenase | 1.62 | 0.02 | up |
|  | Dmahothocarpusptg000001lG011450 | putative 6-phosphogluconate dehydrogenase | 1.28 | 0.05 | up |
| Peroxidase and catalase | Dmahothocarpusptg000003lG008280 | putative cytochrome c peroxidase | 3.57 | 0.01 | up |
|  | Dmahothocarpusptg000001lG008880 | cytochrome c peroxidase, mitochondrial | 1.74 | 0.02 | up |
|  | Dmahothocarpusptg000006lG010860 | putative peroxiredoxin tsa1 | 1.38 | 0.04 | up |
|  | Dmahothocarpusptg000002lG018970 | putative catalase | -2.24 | 0.00 | down |
| Thioredoxin and thioredoxin reductase | Dmahothocarpusptg000004lG020290 | phosphoadenosine phosphosulfate reductase | 3.65 | 0.00 | up |
| Other down-regulated genes related to oxidoreductase activity and oxidation-reduction process | Dmahothocarpusptg000004lG006480 | putative choline dehydrogenase | -5.97 | 0.00 | down |
|  | Dmahothocarpusptg000005lG004380 | putative aldehyde reductase 1 | -4.69 | 0.00 | down |
|  | Dmahothocarpusptg000007lG003750 | cytochrome P450 | -4.56 | 0.00 | down |
|  | Dmahothocarpusptg000008lG004740 | putative benzoate 4-monooxygenase cytochrome p450 | -4.29 | 0.00 | down |
|  | Dmahothocarpusptg000004lG030500 | putative fad linked oxidase | -4.36 | 0.00 | down |
|  | Dmahothocarpusptg000001lG018490 | putative alcohol dehydrogenase | -3.52 | 0.01 | down |
|  | Dmahothocarpusptg000006lG010110 | putative l-threonine 3-dehydrogenase | -1.82 | 0.01 | down |
|  | Dmahothocarpusptg000006lG020890 | putative aldo-keto | -3.35 | 0.01 | down |
|  | Dmahothocarpusptg000004lG025600 | Dimethyl-sulfide monooxygenase | -3.67 | 0.01 | down |
|  | Dmahothocarpusptg000008lG012550 | putative tpa: cytochrome | -3.31 | 0.01 | down |
|  | Dmahothocarpusptg000007lG003310 | fad dependent oxidoreductase | -3.88 | 0.01 | down |
|  | Dmahothocarpusptg000006lG015810 | putative restculine oxidase precursor | -3.73 | 0.01 | down |
|  | Dmahothocarpusptg000001lG013380 | putative pisatin demethylase | -3.58 | 0.02 | down |
|  | Dmahothocarpusptg000008lG012520 | putative benzoate 4-monooxygenase cytochrome p450 | -3.39 | 0.02 | down |
|  | Dmahothocarpusptg000006lG020570 | butyryl-CoA dehydrogenase | -2.75 | 0.03 | down |
|  | Dmahothocarpusptg000006lG000330 | isotrichodermin C-15 hydroxylase | -3.10 | 0.03 | down |
|  | Dmahothocarpusptg000004lG015110 | putative GMC oxidoreductase | -2.59 | 0.04 | down |
